# Parental knowledge, attitudes, satisfaction and decisional conflict regarding whole genome sequencing in the Genomic Medicine Service: a multisite survey study in England

**DOI:** 10.1136/jmg-2024-110458

**Published:** 2025-02-12

**Authors:** Ria Patel, Bettina Friedrich, Saskia C Sanderson, Holly Ellard, Celine Lewis

**Affiliations:** 1 UCL Medical School, University College London, London, UK; 2 Population, Policy and Practice, UCL GOS Institute of Child Health, London, UK; 3 UCL Institute of Epidemiology and Health Care, London, UK; 4 Department of Biostatistics & Health Informatics, SGDP Centre, Institute of Psychiatry, Psychology and Neuroscience, King's College London, London, UK; 5 Department of Behavioural Science and Health, University College London, London, UK; 6 UK Mental Health Mission, London, UK; 7 North Thames Genomic Laboratory Hub, Great Ormond Street Hospital for Children NHS Foundation Trust, London, UK

**Keywords:** Child Health, Evidence-Based Practice, Genetic Testing, Health Services Research, Pediatrics

## Abstract

**Background:**

Whole genome sequencing (WGS) for paediatric rare disease diagnosis is now available as a first-line test for certain clinical indications in the Genomic Medicine Service in England. The aim of this study was to assess decisional conflict regarding WGS at the time of consent as well as parental knowledge, attitudes and satisfaction.

**Methods:**

We conducted a multisite quantitative survey including validated measures. Surveys were sent out across seven National Health Service Trusts in England to parents of children offered WGS, within 4 weeks of their appointment.

**Results:**

374/1366 survey responses were included in the final dataset. Parents were highly satisfied with their WGS appointment (mean=24.47/28), had low decisional conflict (mean=20.09/100) and felt they had received enough information and support to make an informed decision (83.9%). Parents had positive attitudes towards WGS (mean=18.17/20), and those who had discussed WGS with a genetic counsellor or genomic associate had significantly more positive attitudes than those seen by genetic consultants (p<0.001). Most parents (84.3%) strongly agreed (27.2%) or agreed (67.1%) that they had a clear understanding of what a genomic test is. Parents whose child’s condition was reported as more serious (p=0.0011) felt less conflicted about their decision.

**Conclusions:**

The parents in this study had low decisional conflict and most felt they had made an informed decision. Further research after parents receive WGS results to assess whether any, and if so who, regrets their decision, is important.

**WHAT IS ALREADY KNOWN ON THIS TOPIC**

- A diagnosis can have significant clinical as well as psychosocial benefits for patients with rare diseases and their families, but some may later go on to have decisional regret.
- Whole genome sequencing (WGS) has recently been introduced in the National Health Service (NHS) in England as a routine clinical test.
- Families have reported high satisfaction with consent appointments for WGS in clinical research contexts; however, little is known about how effective the consent procedure is for WGS, including how satisfied parents are with their consent appointment.

**WHAT THIS STUDY ADDS**

- It appears that the consent process for WGS for paediatric rare disease diagnosis is effective in that parents are highly satisfied and have low decision conflict.
- A more serious condition in the child was associated with parents experiencing less decisional conflict.
- Participants with higher levels of income, higher education levels or previous experience of genetic testing felt more satisfied with their appointment.

**HOW THIS STUDY MIGHT AFFECT RESEARCH, PRACTICE OR POLICY**

- Genomic associates, a new cadre of health professional in the NHS, appear to be effective in conducting consent conversations for WGS.
- Parents without previous experience of genetic testing and those who had been looking for a diagnosis for a shorter time period were more likely to experience decisional conflict.
- As such, those parents may require more discussion time dedicated to explaining the benefits and limitations of WGS as well as addressing any potential concerns these parents may have regarding testing.

## Background

 Whole genome sequencing (WGS) for paediatric rare disease diagnosis is now available in England as a first-line test for certain clinical indications via the new nationally commissioned Genomic Medicine Service (GMS) of the National Health Service (NHS).^1^ The service, which aims to deliver consolidated, state of the art, high throughput sequencing, capitalises on the infrastructure set up during the 100 000 Genomes Project, a hybrid clinical-research project in which over 70 000 patients (and families) affected by rare disease and cancer had their genomes sequenced.^2^ The NHS is now the first national healthcare system in the world to offer patients genome sequencing in routine clinical care. A key change in the delivery of genetic testing is that certain tests can be ordered by clinicians from subdisciplines outside of genetics in both the primary and secondary care setting (known as ‘mainstreaming’).^3^ In some cases, clinicians are also asked to collect ‘trio tests’, whereby a patients’ genome is sequenced alongside their parents’, which can help to reduce the number of possibly pathogenic variants under consideration and has been shown to increase the chance of a diagnosis.^4^

The diagnostic yield of WGS for previously unsolved rare disease cases has been shown to be up to 55% for certain indications and will therefore have a major impact for rare disease patients, in particular children who are the age group most affected.^5^ A diagnosis can have a significant benefit for paediatric patients and their families in terms of access to management options or clinical trials, prognosis and recurrence risk information, and psychosocial benefits such as access to educational and social support, connecting with other families and relief from guilt.^6^ Nevertheless, research has shown that there may also be negative outcomes from genomic testing, including parents experiencing distress and uncertainty following receipt of sequencing results, frustration and isolation from the lack of information and loss of hope for recovery.^7^ Parents and caregivers (indicated using the term ‘parent’ going forward) who do not receive a diagnostic result may also experience disappointment, distress and loss of hope.^8^ Managing parental expectations of WGS and ensuring parents make informed decisions about testing is therefore key.

Our previous research conducted during the 100 000 Genomes Project highlighted that most participants were satisfied with the consent process and felt they had made an informed decision to take part.^9^ However, around 15% did not feel they had made an informed decision despite agreeing to testing, there were misunderstandings around the limitations of the technology, and around a quarter were concerned around data sharing. Examining the quality of the pretest counselling provided at the time of decision-making is therefore of value, particularly now that the consent discussion is part of routine testing (as opposed to as part of a research study) and may be performed by mainstream clinicians. NHS England and Genomics England have designed a range of patient-facing materials to support parents in the decision-making process such as online educational resources^10 11^ including ‘easy read’^12^ and translated versions,^13^ and a ‘record of discussion’ form^14^ for parents to read and sign, for both them and their child, which covers both the clinical implications of a test as well as an offer (to both the child and the parents) to participate in the National Genomic Research Library (NGRL),^1^ a comprehensive database that enables approved researchers to access deidentified genomic data, health data and samples. However, as the GMS is still in its infancy, little is currently known regarding what parents’ attitudes, understanding and experiences of WGS within a purely clinical context are, including their knowledge and attitude towards WGS, whether they feel they have made an informed decision to undergo testing and whether they are satisfied with the process overall.

Attitude, knowledge and satisfaction with the decision have all been identified as important components of informed decision-making.^15^ It is important to explore these topics prior to parents receiving WGS results to examine the quality of pretest counselling and to understand whether the initial choice to have WGS was informed. Informed decision-making has been defined as one in which an individual has a positive attitude towards undergoing a test, has relevant knowledge about the test and undergoes it (or a negative attitude and declines it) and is generally measured at the time of decision-making, prior to receipt of results, as the results of the test may impact how they viewed.^16^ Moreover, the decision to have WGS is not always straightforward because potential benefits, for example, receiving a diagnosis, need to be weighed against other considerations, for example, family implications and data security.^17^ Our findings could provide valuable insights and guidance into how consent discussions for WGS should be conducted.

This is a longitudinal survey delivered at two time points, T1 within 4 weeks of the consent appointment and prior to receiving WGS results, and T2 after receiving WGS results. The aims of the T1 survey were to assess knowledge, attitudes, satisfaction, decisional conflict and informed decision-making at the time of consent. We also collected data around parental anxiety and parent quality of life, which will be reported in a separate paper. The T2 survey will measure whether the result from WGS impacts parental attitudes and anxiety as well as include measures of clinical utility, parent and child quality of life and family impact.

In this paper, we set out to answer the following four questions:

1. What knowledge of genome sequencing do parents have, and do they feel they have a clear understanding of what a genomic test is?
2. What are parents’ attitudes towards WGS offered in the NHS GMS?
3. Are parents satisfied with the consent procedure and do they experience decisional conflict?
4. Do parents feel they received sufficient information and support to make an informed decision about WGS in the NHS GMS?

## Methods

### Study design

This was a multisite quantitative survey study conducted at two time-points; time 1 (T1) following consent and time 2 (T2) following receipt of results.

### Survey design measures and piloting

The content of the survey was informed: by the research aims; by a previous survey we conducted assessing decision-making, attitudes and understanding among participants in the 100 000 Genomes Project; and in collaboration with our advisory and PPI team.^9 18^ An initial list of measures and specifically designed questions were piloted by CL with the project advisory and PPI team comprising a genetic counsellor, two clinical geneticists, a paediatrician, a rare disease policy advisor, a behavioural scientist, two patient group representatives and two parents of children with a rare genetic condition. Each member was individually interviewed to gather their reflections on the most important measures and questions to include in the survey. We did this by asking participants to rate each measure and set of questions on a scale of 1–5; this enabled us to ascertain which were considered most important and thus reduce the initial list to a manageable size. A second draft of the survey was then designed by CL and BF, and then BF piloted it with three parents of children with a rare disease recruited through a patient organisation as well as an academic with expertise in the field. They were asked to complete the survey, after which BF arranged a call or online meeting to go through the questions and get feedback on length, feasibility, clarity and appropriateness of the wording. Some additions (and removal) of specifically designed questions occurred at this stage as well as minor changes to wording and layout.

### Measures

The final version of the survey included the following measures (see online supplemental materials for the full survey).

*KOGS* was assessed using the 9-item KOGS to measure general knowledge of genomic testing^19^ and 4-item specifically designed true or false questions to measure context-specific knowledge, that is, knowledge related to WGS in the context of the GMS (eg, “Your child’s DNA sample will be destroyed after the analysis has taken place”). We also included one question to assess subjective knowledge (“I have a clear understanding of what a genomic test is” measured on a 5-point scale: strongly disagree to strongly agree).

*Attitude towards genome sequencing* was assessed using a 5-item measure developed in our previous survey used during the 100 000 Genomes Project,^9^ including constructs such as harmful to beneficial, unimportant to important.

*Decisional conflict* was assessed using the 15-item Decisional Conflict Scale.^20^

*Parents’ emotional state* was assessed using the GAD-7 anxiety measure,^21^ the 6-item Brief Resilience Scale^22^ and a single question to assess tolerance for uncertainty (“How well do you feel you deal with uncertainty in your life?”) with five response options ranging from ‘not at all well’ to ‘very well’.

*Satisfaction* was assessed using an amended 7-item *Patient Satisfaction Questionnaire for use in Clinical Genetics Settings* Scale^23^ (amended so it was suitable for use across all clinical settings). A question was also included to ascertain the mode or delivery of appointment (in person, using virtual meeting software, or by telephone).

*Parent characteristics* measures included questions to assess gender, age, relationship to the patient, number of children, educational attainment, household income, ethnicity and religious faith.

*Child characteristics* measures included age, how long they have been looking for a diagnosis and whether they had previous genetic tests.

### Participants and recruitment

Participants in this study were parents of children (16 years and under at time of recruitment) with an undiagnosed rare disease who were attending an appointment at one of seven collaborating NHS sites across England (located in London, the south of England and the north of England) during which they discussed and/or consented to WGS. Appointments took place between May 2022 and August 2023.

Survey packs were sent to participants by their NHS site within 4 weeks of their appointment. The packs contained (1) a paper survey (which included a QR code so that the survey could be completed online), (2) a participant information sheet, (3) a freepost envelope and (4) an invitation letter from the respective NHS site. Returning a survey was taken as implied consent to take part. At three of the seven sites non-responders were followed up either by telephone (two sites) or another survey was sent by post (one site); however, this was not always done consistently. Participants were offered a £10 gift voucher as a token of appreciation for their participation. At the end of the survey participants were invited to leave their contact details so that they could be sent the T2 survey and the gift voucher (£10), and so that a subset could be invited for a follow-up qualitative interview.

For each participant we also collected data from the recruiting sites on the WGS panel requested by the clinician so that we were able to gather information about the patients’ clinical indication (denoted using an ‘R’ number as per the National Genomic Test Directory),^1^ as well as the type of health professional who had consented the parents (divided into three groups: (1) genetic counsellor/genomics associate; (2) genetic consultant or (3) non-genetic consultant). A genomic associate is part of the genetic counsellor career structure and leads on administrative support for the clinic, the patient and the clinical activities of the clinical geneticist and genetic counsellor.^24^

### Sample size

To compare decision regret at T2 between those parents of patients who did versus did not receive a diagnostic result, a minimum of 67 participants was required in both groups to detect a medium effect size (0.5) with an 80% power level. As diagnostic rates using genomic testing are currently around 40% when trio-based analysis is performed,^4^ a minimum of 168 participants was required. To account for dropout between the T1 and T2 survey, which was around 50% in previous research,^9^ we therefore aimed to recruit around 400 participants at T1.

### Analysis

1. *What knowledge of genome sequencing do parents have, and do they feel they have a clear understanding of what a genomic test is?*
  To evaluate knowledge and understanding of WGS, subjective knowledge, objective knowledge (KOGS) and context-specific knowledge scores were analysed and reported using descriptive statistics. To explore relationships between subjective and objective knowledge (KOGS), Kendall’s tau-b was used to assess ordinal associations.
  To evaluate relationships between participant characteristics and objective and context-specific knowledge scores, bivariate analyses were used. For dichotomous variables (eg, previous genetic test), Mann-Whitney U tests were used and for ordinal variables (eg, time spent looking for a diagnosis), Kendall’s tau-b was used.
2. *What are parents’ attitudes towards WGS offered in the NHS GMS?*
  Overall attitude scores were analysed using descriptive statistics. The relationship between attitude scores and the type of healthcare professional seen (genetic counsellor/genomic associate versus genetic consultant) was analysed using an independent samples t-test. Effect size was calculated using Cohen’s d to measure the magnitude of this effect.
3. *Are parents satisfied with the consent procedure and do they experience decisional conflict?*
  Satisfaction scores were analysed using descriptive statistics. For both satisfaction and decisional conflict, Kendall’s tau-b was used to assess correlations with ordinal variables such as resilience scores or household income, while Mann-Whitney U tests were used for dichotomous variables such as previous genetic tests.
4. *Do parents feel they received sufficient information and support to make an informed decision about WGS in the NHS GMS?*
  The informed decision measure was analysed using descriptive statistics. Associations between the informed decision measure and objective knowledge scores (KOGS) were assessed using Kendall’s tau-b.

Given the number of comparisons (n=21), to control for the risk of type I error, the Bonferroni adjustment was employed, resulting in a corrected alpha level of 0.003. In the results, we highlight those that remained statistically significant at the adjusted p value threshold.

## Results

### Sociodemographic characteristics and decision-making of participants

In total, 1366 surveys were distributed across 7 NHS Trusts in England with 383 returned. Of those returned, seven were excluded because of missing data (they only included demographic data and the remaining items had not been completed) and two were excluded because they were duplicated surveys (n=2) where the same responder had completed the survey two times, leaving 374 surveys including in the final analysis (27% response rate).

The majority of survey respondents were female gender (89%), biological parent (91.7%), White or White British ethnicity (81.5%), and had consented to WGS (94.7%) as well as the NGRL (68.4%). Respondent characteristics are summarised in tables1  2, along with details about their decision-making.

**Table 1:** Participant characteristics

Characteristic	Response options	N (%)
Participant type	Biological parent	343 (91.7)
	Non-biological parent	17 (4.5)
	Carer	5 (1.3)
	Other	3 (0.8)
	Missing	6 (1.6)
Gender	Female	333 (89)
	Male	38 (10.2)
	Another gender identity	1 (0.3)
	Missing	2 (0.5)
Age, years	Mean (SD), range	37.98 (7.73) 21–77
Number of children	Mean (SD), range	2.31 (1.23) 1–9
Education	No qualification	18 (4.8)
	GCSE or O level	63 (16.8)
	GCE, A-level or similar	37 (9.8)
	Vocational (BTEC/NVQ/diploma)	60 (16)
	Bachelor’s degree or equivalent	101 (27)
	Master’s degree or equivalent	50 (13.4)
	PHD, MD or equivalent	9 (2.4)
	Missing	36 (9.6)
Household income	Below £10 000	31 (8.2)
	£10 001 to £30 000	95 (25.4)
	£30 001 to £50 000	71 (19)
	£50 001 to £70 000	61 (16.3)
	Over £70 001	61 (16.3)
	Missing	55 (14.7)
Ethnicity	Asian or Asian British	32 (8.6)
	Black or black British	8 (2.1)
	Mixed	6 (1.6)
	White or white British	305 (81.5)
	Other ethnic group	11 (2.9)
	Missing	12 (3.2)
Religion	None	191 (51)
	Buddhist	1 (0.3)
	Christian/Catholic	142 (38)
	Hindu	1 (0.3)
	Jewish	4 (1.1)
	Muslim	27 (7.2)
	Sikh	3 (0.8)
	Other	4 (1.1)
	Missing	1 (0.3)
Did parent consent to WGS	Yes	354 (94.7)
	No	4 (1.1)
	Missing	16 (4.3)
Did parent consent to NGRL	Yes	256 (68.4)
	No	14 (3.7)
	Was not asked	16 (4.3)
	Don’t know	85 (22.7)
	Missing	3 (0.8)
Type of health professional that consented parent	Genomic associate, genetic counsellor or nurse	157 (42)
	Clinical geneticist or genetics registrar	146 (39)
	Non-genetic consultant	14 (3.7)
	Information not available	33 (8.8)
	Not consented	5 (1.3)
	Missing	19 (5.1)
Mode of appointment	In person	251 (67.1)
	By telephone	76 (20.3)
	Virtual	20 (5.3)
	Missing	27 (7.2)

NGRL, National Genomic Research Library; WGS, whole genome sequencing.

**Table 2:** Child characteristics

Characteristic	Response options	N (%)
Condition type	Cardiology	3 (1.4)
	Developmental disorders	199 (68.4)
	Endocrinology	5 (2.4)
	Haematology	1 (0.5)
	Immunology	7 (3.3)
	Metabolic	11 (5.2)
	Mitochondrial	5 (1.3)
	Musculoskeletal	11 (5.2)
	Neurology	55 (23.2)
	Ophthalmology	19 (9)
	Renal	3 (1.4)
	Ultrarare and atypical monogenic disorders	10 (4.8)
Age, years	Mean (SD), range	6.2 (4.3), 0–17
Previous genetic tests?	Yes	194 (51.9)
	No	160 (42.8)
	Don’t know	14 (3.7)
	Missing	6 (1.6)
Length of time looking for a diagnosis, years	Mean (SD), range	4.04 (2.48), 1–8
My child’s condition is serious	Strongly agree	66 (17.6)
	Agree	120 (32.1)
	Neither agree nor disagree	126 (33.7)
	Disagree	47 (12.6)
	Strongly disagree	13 (3.5)
	Missing	2 (0.5)
My child’s condition has major consequences on their life	Strongly agree	105 (28.1)
	Agree	127 (34)
	Neither agree nor disagree	90 (24.1)
	Disagree	34 (9.1)
	Strongly disagree	13 (3.5)
	Missing	5 (1.3)

### Parents’ understanding of WGS including how it is being offered in the NHS GMS

**Figure 1:**
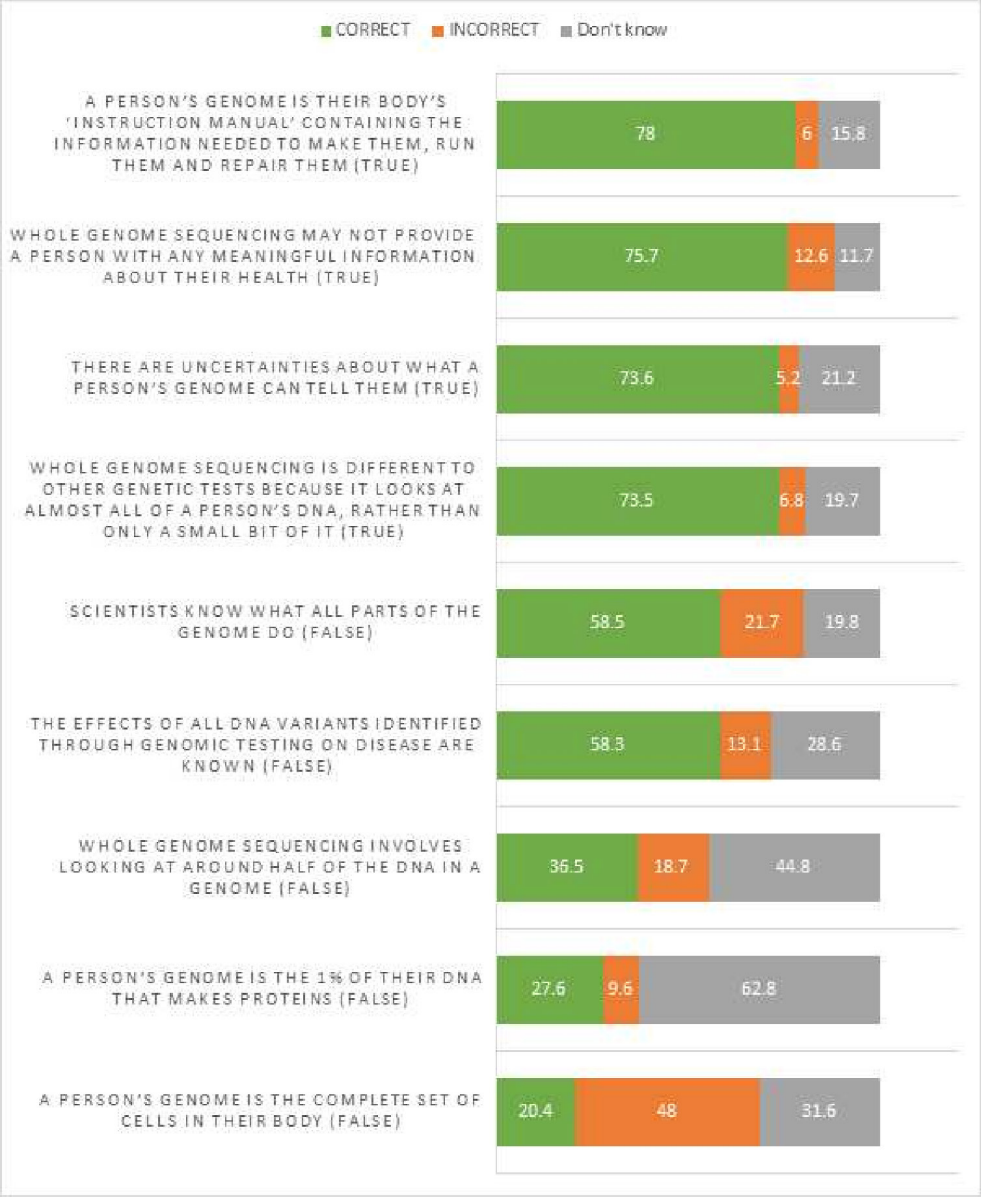
Knowledge of genome sequencing scale items.

Most parents (84.3%) either strongly agreed (27.2%) or agreed (57.1%) that they had a clear understanding of what a genomic test is, indicating that subjective knowledge was high. Only 8 (2.2%) disagreed, 7 (1.9%) strongly disagreed and 40 (11%) neither agreed nor disagreed. For general knowledge of WGS using the KOGS, overall mean score was 5.03 out of 9 (n=352, SD=2.13). Figure 1 shows the response percentages for each item in the scale.

In bivariate analysis, the KOGS score was higher among parents who had higher subjective knowledge (τ_b_=0.091, p=0.039). The mean KOGS score for those who agreed or strongly agreed they had good knowledge about WGS was 5.21 (SD=2.04) compared with a mean score of 4.87 (SD=1.89) among those that disagreed or strongly disagreed they had good knowledge about WGS.

Respondents with high educational attainment scored higher KOGS scores than those with low educational attainment (Z=−3.196, p=0.001). KOGS scores were also significantly associated with the length of time parents had been looking for a diagnosis (τ_b_=0.099, p=0.015), and previous experience of genetic testing (U=12 135, z=−2.124, p=0.034), with parents who had been looking for a diagnosis longer and/or had had previous genetic tests having a better understanding of WGS. KOGS scores were not associated with any other variables. After Bonferroni correction, only the association between participants’ KOGS score and educational attainment remained significant.

### Participants’ context-specific knowledge

**Figure 2:**
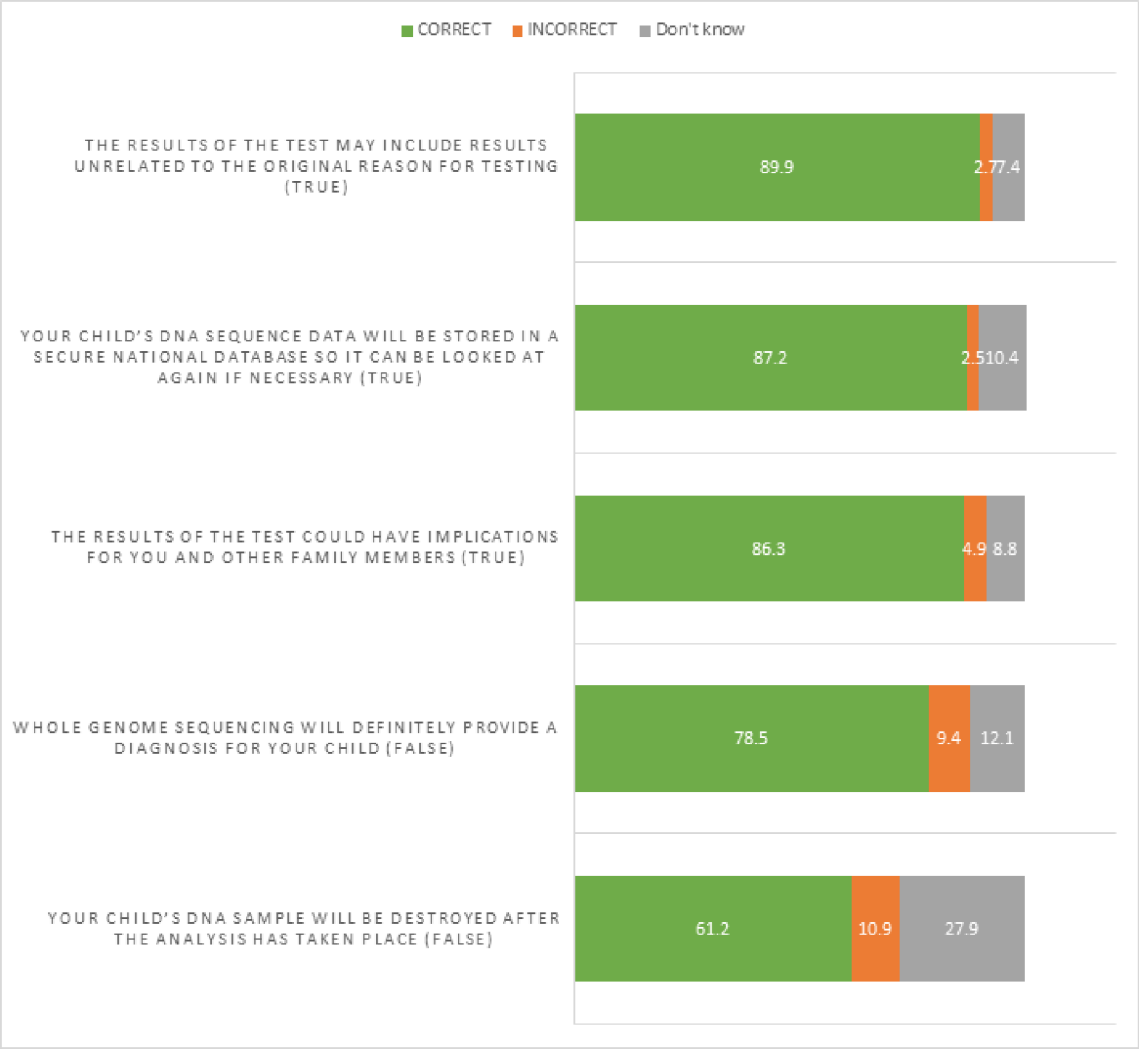
Context-specific knowledge.

The mean context-specific knowledge score was 4.03/5 (n=357, SD=1.21). Figure 2 shows the response percentages for each item in the scale. Participants were most unsure about whether their child’s DNA sample would be destroyed after analysis (item 3).

Mean context-specific knowledge scores were higher among participants who self-reported as white ethnicity than groups other than white (U=9764.5, p=0.003). The longer participants had been looking for a diagnosis and those who had had previous genetic testing also scored higher (τ_b_=0.16, p<0.001 and U=11 383, z=−3.606, p<0.001, respectively). Context-specific knowledge was not associated with any other variables. After Bonferroni correction, the association between both length of time spent looking for a diagnosis and previous genetic tests remained significant.

### Parents’ attitudes towards WGS offered via the NHS GMS

The mean attitude score of participants was 18.17/20 (n=343, SD=2.96) with higher scores indicating more positive attitudes towards WGS. Participants who had been consented by either a genetic counsellor or genomic associate had higher attitude scores compared with those consented by a genetic consultant (mean 18.83 vs 17.29; p<0.001); with a moderate-to-large effect size (Cohen’s d=0.52, 95% CI (0.29, 0.75)). This remained significant after Bonferroni correction. There were no significant differences in participants’ attitude scores between those seen by genetic versus non-genetic consultants.

### Parents’ satisfaction with the consent procedure and decisional conflict

#### Satisfaction

Overall, parents were very satisfied with their consent appointment with the mean satisfaction score being 24.47/28 (n=363, SD=4.67). Satisfaction was significantly associated with subjective informed choice (τ_b_=−0.163, p<0.001), indicating that the more informed participants felt, the more satisfied they were with the appointment. Satisfaction was not associated with the type of healthcare professional that consented them or the mode of delivery of the appointment, but was associated with parent resilience (τ_b_=0.104, p=0.009), suggesting the more resilient parents felt, the more satisfied they were with the appointment. Satisfaction was positively associated with household income (τ_b_=0.176, p<0.001), education (τ_b_=0.089, p=0.039) and previous genetic testing (U=17 773, z=3.395, p<0.001), suggesting that participants with higher levels of income, higher education levels or previous experience of genetic testing felt more satisfied with their appointment. After Bonferroni correction, the associations of satisfaction with subjective informed choice, income and previous genetic testing remained significant.

#### Decisional conflict

The mean decisional conflict score was low (mean=20.09, range 0 (no decisional conflict) to 100 (high decisional conflict); n=349, SD=16.5). There was a significant correlation between attitude score and decisional conflict score (n=342, τ_b_=−0.217, p<0.001), indicating that the less conflicted participants felt about WGS, the more positive their attitude was. There was a correlation between decisional conflict and subjective knowledge (n=344, τ_b_=−0.317, p<0.001) indicting that those who did not feel they had a clear understanding of WGS had more decisional conflict. Decisional conflict was also associated with objective knowledge measures (KOGS: n=333, τ_b_= −0.9, p=0.039; Context-specific knowledge scale: n=337, τ_b_=−0.124, p=0.003) indicating that parents with less knowledge about WGS were more conflicted about whether to consent to WGS.

Regarding the parents’ characteristics, those with higher resilience scores had significantly lower decisional conflict scores (τ_b_=−0.1, p=0.01), indicating that the more resilient participants were, the less conflicted they felt about their decision. Regarding the child’s characteristics, decisional conflict was associated with the seriousness of the child’s condition (τ_b_=0.105, p=0.011) indicating that the more serious the child’s condition was, the less conflicted parents felt. Similarly, decisional conflict was associated with previous genetic testing experience (U=11 808.5, z=−2.085, p=0.037), with parents whose child had had previous experience of genetic testing less conflicted. After Bonferroni correction, only the associations between decisional conflict and attitude score with subjective knowledge scores remained significant.

### Parents’ views on informed decision-making about WGS

Most parents, 308/367 (83.9%) said yes, they had received enough information and support from their healthcare team to make an informed decision about WGS; 48 (13.1%) said partly, 4 (1.1%) said no and 7 (1.9%) were not sure. Informed choice was associated with the KOGS score (τ_b_=−0.109, p=0.017), suggesting that the better parents’ general knowledge of WGS was, the more likely they were to feel they had made an informed decision. However, this did not remain significant after Bonferroni adjustment.

## Discussion

In this study, we found that among survey responders, the NHS GMS consent process for genome sequencing appears to be working effectively: most participants reported that they had a clear understanding of what a genomic test is, and had adequate knowledge particularly around the use of WGS in the GMS. Moreover, participants’ attitudes were largely positive towards WGS; they were also highly satisfied with the consent procedure, experienced low decisional conflict and felt they received sufficient information and support to make an informed choice. These results are encouraging and are perhaps reflective of the significant work conducted by NHS England to upskill the workforce to effectively facilitate genomic testing.^25^

At the same time, these results need to be interpreted with some degree of caution, first because the vast majority of our participants were seen by genomic specialists, with very few participants counselled by mainstream clinicians, and second because of the low response rate, limiting the generalisability of the findings. Further research with participants who have been counselled and consented by non-genetic specialists is therefore important, particularly given the importance of the move towards mainstreaming in the NHS.^26^ Moreover, while the majority of parents in our sample reported low decisional conflict, the benefits and risks of offering WGS in clinical practice are not fully characterised as the use of the technology is still in its infancy with research around clinical and psychosocial outcomes only now beginning to emerge.^27^,^29^ It may be that the finding implies an effective consent process, but it could also suggest that the consent process minimises the true uncertainty about benefits, risks and limitations. Further (behavioural) research when patients receive their results is therefore vital to improve our understanding in this area.

We explored whether there were differences in knowledge, attitudes, satisfaction and decisional conflict between parents who were consented by a genomic associate or genetic counsellor versus a clinical geneticist; few differences were detected. Genomic associates are increasingly being employed within the GMS to support the consent process for WGS including conducting the consent conversation itself.^24 30^ Other studies being conducted within this programme of work have also highlighted the effectiveness of employing genomic associates to take on this consenting role. In a content analysis of the paediatric WGS consent conversation, it was found that genomic associates covered on average more topics around WGS (and the NGRL) than consultants and their appointments were also on average longer.^31^ In another study mapping the consent process for WGS in which qualitative interviews with health professionals were conducted, employing genomic associates was identified as an efficient way to tackle existing waiting lists as they can take on the bulk of the administrative tasks associated with the consent process (form filling, uploading documents, chasing blood samples, etc) thus freeing up time for consultants to focus on routine clinical genetics appointments.^32^ This growing body of evidence points to the important role that this relatively new cadre of health professional has in supporting WGS in the GMS.

We found that, in this sample of parents, those who had been looking for a diagnosis for a shorter time period were more likely to experience decisional conflict. This tentatively suggests these parents may require more discussion time dedicated to explaining the benefits and limitations of WGS as well as addressing any potential concerns they may have regarding testing. This may be particularly pertinent for parents being offered WGS in the mainstream setting, where it may be less likely that they would have encountered genetic testing previously. Embedding genetic counsellors into mainstream settings, which is already beginning to happen in practice, may be beneficial as they are trained to explain complex genetic information as well as support patients and families to make informed decisions.^33^ In addition, those with lower resilience were also found to have greater decision conflict. Talking through the various outcomes from WGS and how these parents might feel if they were to receive any of them might be helpful for this group of parents.

In this study, we found that most parents (84%) said they had received enough information and support to make an informed decision about WGS. However, around a quarter of our participants did not correctly understand that they might not get a diagnosis from WGS, a key aspect of WGS that is considered important for informed decision-making.^34 35^ This could indicate an area that those conducting WGS consent conversations should check that parents understand, as we know that unmet expectations from genomic testing are linked with negative experiences.^36^ However, the relationship between knowledge and the concept of informed decision-making is a thorny one. Authors of a recent study conducted in Germany and Switzerland have pointed to WGS consent conversations as being potentially problematic because of the enormous amount of relevant information given and the variety and complexity of the possible test outcomes, and question whether consent can be truly informed.^35^ Others have recommended a move towards the phrase ‘appropriately’ rather than ‘fully’ informed consent in genetics and argue that appropriate consent can be achieved if the process promotes autonomy, well-being and trust in medicine.^37^

### Limitations

We were unable to collect the demographic data and condition types of non-responders, so we cannot determine how representative our sample is of parents undergoing WGS more broadly. Responders with more favourable attitudes towards WGS may have been more motivated to take the time and effort to respond. Nevertheless, mean attitude scores (18.2/20) were comparable to those reported by participants taking part in the 100 000 Genomes Study (18.4/20) lending some validity to the present findings.^9^ Moreover, our sample was primarily of white ethnicity, well educated and relatively affluent. A further limitation of our study therefore is that it does not necessarily reflect the experiences of more marginalised, historically underserved communities who experience additional barriers in accessing clinical care.^38^ This may also be partly because we were unable to provide the survey translated into languages other than English, in part because many of the measures used have not been validated in other languages. Our response rate was also low (27%), and we had a relatively small number of black/black British (2%) and Asian/Asian British (8.1%) parents participate. Research has shown that many barriers exist to ethnic minority populations participating in genomic research, including perceptions of research benefits, language difficulties, mistrust in research and confidentiality concerns.^39^ Further research with these groups, informed through competence frameworks^40^ to recruit and engage participants, is vital if we want to understand whether the consent process for WGS is effective and equitable. Finally, due to the length of the survey, we did not explore decision-making around the NGRL which is typically sought in the same appointment. Future research would valuably investigate parents’ decisional conflict, knowledge and attitudes towards their sample being used for research purposes in addition to clinical purposes.

## Conclusion

Our findings suggest that, at least among those who responded to the survey, parents making decisions about WGS in the relatively new GMS feel satisfied with the consent process. However, our results need to be interpreted with some degree of caution due to the low response rate and mainly white and well-educated sample, emphasising the need for research around the experience of WGS across diverse groups to understand whether certain populations require more resources during decision-making. Further research with parents once they receive their WGS results—whether it be a diagnostic, negative or inconclusive result—will shed more light on the clinical, psychological, behavioural and social impacts and utility of WGS, as well as on the processes of consent and results return.

## Supplementary material

10.1136/jmg-2024-110458 online supplemental file 1

## Data Availability

Data are available upon reasonable request.
