# Supplementary material for: Parental knowledge, attitudes, satisfaction and decisional conflict regarding whole genome sequencing in the Genomic Medicine Service: a multisite survey study in England
Source: J Med Genet. 2025 Feb 12;62(4):e110458. doi: 10.1136/jmg-2024-110458 (PMC12015048; doi:10.1136/jmg-2024-110458)
Supplement: online supplemental file 1 [file jmg-62-4-s001.docx]

[Survey T1: Version 1.0 date: 09/05/2022]

Survey ID: R number:

**THE PARENT EXPERIENCE SURVEY – PART 1**

**Why am I being invited to take part?** We are inviting you to take part in this survey as you are the parent of a child with an **undiagnosed condition** who is being offered **a genomic test** to try and find a diagnosis.

Recently, the NHS began offering a new type of test (called a genomic test or genome sequencing) to improve the way we diagnose children with undiagnosed conditions. Currently, we know very little about what it is like for parents when their child goes through this testing process. We also know very little about the impact the result has on the child’s as well as the parent’s life. Through this survey, we hope to build a picture of parents’ experiences which we can use to improve how we offer these tests and support parents in the future.

**What will happen if I take part?** This is the first of **2 surveys** that you will be sent during the study. You will receive a second survey around 12 months after completing the first. This will help us to capture information about you and your child’s journey throughout the testing process.

Each survey will take about 20 minutes to complete. All participants who return a completed survey will be offered a **£10 Amazon voucher** as a token of our appreciation for taking part **(£20 for completing both)**.

**Who should complete the survey?** The survey should be completed by the parent who attended the consent appointment for a genomic test. If more than one parent attended, ideally, the child’s main caregiver should complete the survey. The study works best if the same person completes both surveys.

**How do I complete the survey?** There are a number of ways you can complete this survey:

1. On paper and return it in the freepost envelope enclosed

2. Online using the following link: **LINK TO SURVEY**

3. On your phone using the following QR code

QR CODE HERE

4. Call us and we can read out the survey over the phone: xxxxx

5. Text to let us know you are interested: **xxxxxx**

Some of the questions we ask may seem repetitive or you may not always understand why we might be asking certain questions. However, many of these questions are ‘tried and tested’, and have been used in other important studies. Using the same questions therefore allows us to compare responses across different studies. Please therefore answer as many as you can, as best you can.

Thank you very much for taking the time to complete this survey. Your answers to these questions are incredibly important to us. We **really appreciate** you taking the time to take part in this study.

**Section 1: Attitudes**

This section is about your thoughts and feelings about genomic testing

| \| For each of the following four questions, please circle the number from 1 to 5 on the scale that best describes how you feel at the moment. \| \| --- \|   **1.** For me and my child, having a genomic test is:   \| Harmful \|  \|  \|  \| Beneficial \| \| --- \| --- \| --- \| --- \| --- \| \| 1 \| 2 \| 3 \| 4 \| 5 \|   **2.** For me and my child, having a genomic test is:   \| \| Unimportant \|  \|  \|  \| Important \| \| --- \| --- \| --- \| --- \| --- \| \| 1 \| 2 \| 3 \| 4 \| 5 \| \|  \|  \|  \|  \|  \|  \| \| --- \| --- \| --- \| --- \| --- \| --- \| --- \| --- \| --- \| --- \| --- \| --- \| --- \| --- \| --- \| --- \| --- \|   **3.** For me and my child, having a genomic test is:   \| A bad thing \|  \|  \|  \| A good thing \| \| --- \| --- \| --- \| --- \| --- \| \| 1 \| 2 \| 3 \| 4 \| 5 \|   **4.** For me and my child, having a genomic test is:   \| \| Not helpful \|  \|  \|  \| Helpful \| \| --- \| --- \| --- \| --- \| --- \| \| 1 \| 2 \| 3 \| 4 \| 5 \| \|  \|  \|  \|  \|  \|  \|  \| \| --- \| --- \| --- \| --- \| --- \| --- \| --- \| --- \| --- \| --- \| --- \| --- \| --- \| --- \| --- \| --- \| --- \| --- \| |
| --- | --- | --- | --- | --- | --- | --- | --- | --- | --- | --- | --- | --- | --- | --- | --- | --- | --- | --- | --- | --- | --- | --- | --- | --- | --- | --- | --- | --- | --- | --- | --- | --- | --- | --- | --- | --- | --- | --- | --- | --- | --- | --- | --- | --- | --- | --- | --- | --- | --- | --- | --- | --- | --- | --- | --- | --- |

**Section 2: Knowledge**

| This section is about how you rate your understanding of genomic testing.  Please indicate whether you agree or disagree with the following statement by ticking the appropriate box:   \|  \| Strongly disagree \| Disagree \| Neither agree nor disagree \| Agree \| Strongly agree \| \| --- \| --- \| --- \| --- \| --- \| --- \| \| **1.** I have a clear understanding of what a genomic test is \| □ \| □ \| □ \| □ \| □ \| |
| --- | --- | --- | --- | --- | --- | --- | --- | --- | --- | --- | --- | --- |

This section is about your actual understanding of genomic testing.

These questions help us to understand whether we are doing a ‘good job’ of explaining the test.

For each of the following statements, please indicate whether you think each statement is “True” or “False” by ticking the appropriate box.

We know some of these questions are difficult. If you don’t know or are not sure, this is absolutely fine -- please just tick the “Don’t know” box.

*Sanderson, S.C., et al., Development of the Knowledge of Genome Sequencing (KOGS) questionnaire. Patient Educ Couns, 2018. 101(11): p. 1966-1972.*

|  | **True** | **False** | **Don’t know** |
| --- | --- | --- | --- |
| **2.** A person’s genome is their body’s ‘instruction manual’ containing the information needed to make them, run them and repair them |  |  |  |
| **3.** Scientists know what all parts of the genome do |  |  |  |
| **4.** There are uncertainties about what a person’s genome can tell them |  |  |  |
| **5.** Whole genome sequencing may not provide a person with any meaningful information about their health |  |  |  |
| **6.** A person’s genome is the complete set of cells in their body |  |  |  |
| **7.** Whole genome sequencing involves looking at around half of the DNA in a genome |  |  |  |
| **8.** A person’s genome is the 1% of their DNA that makes proteins |  |  |  |
| **9.** The effects of all DNA variants identified through genomic testing on disease are known |  |  |  |
| **10.** Whole genome sequencing is different to other genetic tests because it looks at almost all of a person’s DNA, rather than only a small bit of it |  |  |  |
| **11.** Whole genome sequencing will definitely provide a diagnosis for your child |  |  |  |
| **12.** The results of the test may include results unrelated to the original reason for testing |  |  |  |
| **13.** Your child’s DNA sample will be destroyed after the analysis has taken place |  |  |  |
| **14.** Your child’s DNA sequence data will be stored in a secure national database so it can be looked at again if necessary |  |  |  |
| **15.** The results of the test could have implications for you and other family members |  |  |  |

**Section 3: Your decision about genomic testing**

In this section, we are interested in knowing what your feelings are about your child having a genomic test

**1.** Did you feel you had enough information and discussion with doctors or other healthcare providers to make an informed choice about your child having a genomic test?

| Yes |  |
| --- | --- |
| Partly |  |
| No |  |
| Not sure |  |

**2.** Which of the following options did you choose? Please tick one.

**□** **Option 1:** I chose for my child to have a genomic test

**□** **Option 2:** I chose for my child **not** to have a genomic test

*O'Connor, A.M., Validation of a decisional conflict scale. Med Decis Making, 1995. 15(1): p. 25-30.*

Please now answer the following questions about your decision.

|  | Strongly disagree | Disagree | Neither agree nor disagree | Agree | Strongly agree |
| --- | --- | --- | --- | --- | --- |
| **3.** I know which options were available to me | □ | □ | □ | □ | □ |
| **4.** I know the benefits of each option | □ | □ | □ | □ | □ |
| **5.** I know the risks of each option | □ | □ | □ | □ | □ |
| **6.** I am clear about which benefits matter most to me | □ | □ | □ | □ | □ |
| **7.** I am clear about which risks matter most | □ | □ | □ | □ | □ |
| **8.** I am clear about which is more important to me (the benefits or the risks) | □ | □ | □ | □ | □ |
| **9.** I had enough support from others to make a choice | □ | □ | □ | □ | □ |
| **10.** I chose without pressure from others | □ | □ | □ | □ | □ |
| **11.** I had enough advice to make a choice | □ | □ | □ | □ | □ |
| **12.** I am clear about the best choice for my child | □ | □ | □ | □ | □ |
| **13.** I felt sure about what to choose | □ | □ | □ | □ | □ |
| **14.** This decision was easy for me to make | □ | □ | □ | □ | □ |
| **15.** I feel I made an informed choice | □ | □ | □ | □ | □ |
| **16.** My decision shows what is important to me | □ | □ | □ | □ | □ |
| **17.** I expect to stick with my decision | □ | □ | □ | □ | □ |
| **18.** I am satisfied with my decision | □ | □ | □ | □ | □ |

**19**. If you chose to have genomic testing, have all requested blood samples been taken to send to the laboratory for analysis?

**□** **Option 1:** Yes

**□** **Option 2:** No

**□ Option 3:** I don’t know

**20.** If you chose to have genomic testing, did you also agree to take part in the National Genomic Research Library?

**□** **Option 1:** Yes

**□** **Option 2:** No

**□ Option 3:** I was not asked

**□ Option 4:** I don’t know

| **Section 4: How you feel** |
| --- |

*Spitzer, R.L., et al., A brief measure for assessing generalized anxiety disorder: the GAD-7. Arch Intern Med, 2006. 166(10): p. 1092-7*

These next set of questions are about how you are currently feeling.

**There are no right or wrong answers! There is no value judgment. Just respond with the first answer that comes to your mind.**

Over **the last 2 weeks** how often have you been bothered by the following problems?

|  | Not at all | Several days | More than half the days | Nearly every day |
| --- | --- | --- | --- | --- |
| **1.** Feeling nervous, anxious or on edge | □ | □ | □ | □ |
| **2.** Not being able to stop or control worrying | □ | □ | □ | □ |
| **3.** Worrying too much about different things | □ | □ | □ | □ |
| **4.** Trouble relaxing | □ | □ | □ | □ |
| **5.** Being so restless that it is hard to sit still | □ | □ | □ | □ |
| **6.** Becoming easily annoyed or irritable | □ | □ | □ | □ |
| **7.** Feeling afraid as if something awful might happen | □ | □ | □ | □ |

*Smith, B.W., et al., The brief resilience scale: assessing the ability to bounce back. Int J Behav Med, 2008. 15(3): p. 194-200.*

How much do you agree or disagree with the following statements?

|  | Strongly disagree | Disagree | Neutral | Agree | Strongly agree |
| --- | --- | --- | --- | --- | --- |
| **8**. I tend to bounce back quickly after hard times | □ | □ | □ | □ | □ |
| **9**. I have a hard time making it through stressful events | □ | □ | □ | □ | □ |
| **10**. It does not take me long to recover from a stressful event | □ | □ | □ | □ | □ |
| **11**. It is hard for me to snap back when something bad happens | □ | □ | □ | □ | □ |
| **12**. I usually come through difficult times with little trouble | □ | □ | □ | □ | □ |
| **13**. I tend to take a long time to get over set-backs in my life | □ | □ | □ | □ | □ |

|  | Not at all well | Not well | Neutral | Well | Very well |
| --- | --- | --- | --- | --- | --- |
| **14**. How well do you feel you deal with uncertainty in your life | □ | □ | □ | □ | □ |

| **Section 5: About your child’s condition** |
| --- |

Children in the families who participate in our study have a wide range of strengths and difficulties. As such, we have chosen questions that are designed for parents with children of all abilities. At times, you may feel that the questions do not apply to your child. In these instances, we encourage you to answer to the best of your knowledge, even if it feels like your answer is a “best guess” or you are not absolutely certain. All the information you provide us with is extremely valuable.

This section is about your child so we can understand more about the impact their condition on their life.

We are referring here to the condition for which they have been referred for genomic testing.

| **1**. What age is your child currently? |  |  |
| --- | --- | --- |

**2**. How long have you been looking for a diagnosis?

| Less than 1 year |  | 4-5 years |  |
| --- | --- | --- | --- |
| 1-2 years |  | 5-6 years |  |
| 2-3 years |  | 6-7 years |  |
| 3-4 years |  | More than 7 years |  |

**3**. Has your child had previous genetic tests before being offered this one?

| Yes |  |
| --- | --- |
| No |  |
| I don’t know |  |

|  | Strongly disagree | Disagree | Neither agree nor disagree | Agree | Strongly agree |
| --- | --- | --- | --- | --- | --- |
| **4.** My child’s condition is serious | □ | □ | □ | □ | □ |
| **5.** My child’s condition has major consequences on their life | □ | □ | □ | □ | □ |

The next two questions are about your child’s condition. Please tick the boxes below to let us know how much you agree or disagree with each statement.

**Child health-related quality of life scale [licensed scale]**

*Wille N, Badia X, Bonsel G, Burström K, Cavrini G, Devlin N, Egmar AC, Greiner W, Gusi N, Herdman M, Jelsma J, Kind P, Scalone L, Ravens-Sieberer U. Development of the EQ-5D-Y: a child-friendly version of the EQ-5D. Qual Life Res. 2010 Aug;19(6):875-86. doi: 10.1007/s11136-010-9648-y. Epub 2010 Apr 20. PMID: 20405245; PMCID: PMC2892611.*

**Section 6: Impact of the condition on daily life and family**

The following questions help us understand how parents feel about the impact of their child’s undiagnosed condition (which has been the reason for referral) on daily life and on the family. They are important as they help us to understand if and how life changes after genomic testing.

*Grant PE, Pampaka M, Payne K, Clarke A, McAllister M. Developing a short-form of the Genetic Counselling Outcome Scale: The Genomics Outcome Scale. Eur J Med Genet. 2019 May;62(5):324-334. doi: 10.1016/j.ejmg.2018.11.015. Epub 2018 Nov 26. PMID: 30496830.*

Thinking about how much you currently know about your child’s undiagnosed condition, please tick the boxes below to let us know how much you agree or disagree with each statement.

The term 'condition' refers to the undiagnosed difficulties that your child has, that you are hoping to obtain answers about by having a genomic test.

|  | Strongly disagree | Disagree | Neither agree nor disagree | Agree | Strongly agree |
| --- | --- | --- | --- | --- | --- |
| 1. I can explain what the condition means to people outside my family who may need to know (e.g. teachers, social workers) | □ | □ | □ | □ | □ |
| 2. I know who else in my family might be at risk for this condition | □ | □ | □ | □ | □ |
| 3. When I think about the condition in my family, I get upset | □ | □ | □ | □ | □ |
| 4. I know what I can do to change how this condition affects my child | □ | □ | □ | □ | □ |
| 5. I am able to make plans for the future | □ | □ | □ | □ | □ |
| 6. I can make decisions about the condition that may change my future or my child(ren)’s future | □ | □ | □ | □ | □ |

**PedsQL Family Impact Module [Licensed Measure]**

*Varni JW, Sherman SA, Burwinkle TM, Dickinson PE, Dixon P. The PedsQL Family Impact Module: preliminary reliability and validity. Health Qual Life Outcomes. 2004 Sep 27;2:55. doi: 10.1186/1477-7525-2-55. PMID: 15450120; PMCID: PMC521692.*

**Section 7: About your appointment**

Thinking about the consent appointment you had **with the health professional (the person who discussed with you the option of having a genomic test)**, please read each statement and tell us how much you agree with each statement.

|  | Strongly disagree | Disagree somewhat | Uncertain | Agree somewhat | Agree strongly |
| --- | --- | --- | --- | --- | --- |
| **1.** This healthcare professional listened carefully to what I had to say. | □ | □ | □ | □ | □ |
| **2.** This healthcare professional explained things in a way that was easy to understand | □ | □ | □ | □ | □ |
| **3.** I received the information I needed from this healthcare professional | □ | □ | □ | □ | □ |
| **4.** This healthcare professional helped me feel like a partner in care | □ | □ | □ | □ | □ |
| **5.** This healthcare professional spent enough time with me | □ | □ | □ | □ | □ |
| **6**. I was able to share all the necessary information with this healthcare professional | □ | □ | □ | □ | □ |
| **7**. This healthcare professional answered all my questions | □ | □ | □ | □ | □ |

**8**. How was this appointment done?

| In person |  |
| --- | --- |
| Virtually on the computer |  |
| By phone |  |

**Section 8: More about you**

This is the final section. Please answer the following questions about you

**
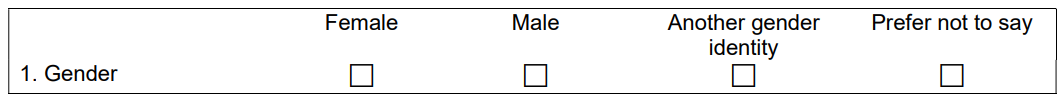
**

**
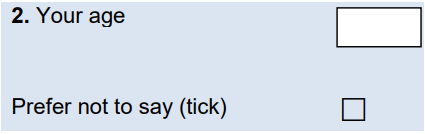
**

**
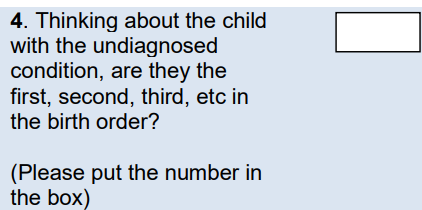
**

**
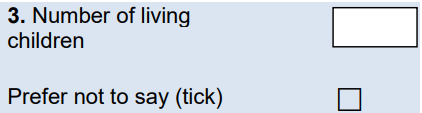
**

**
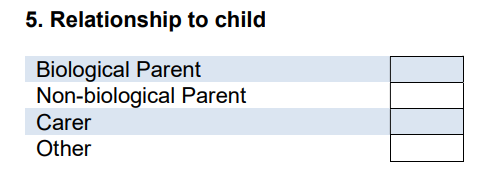
**

**6. Highest qualification 7. Total household income last year**

| No qualification |  |  | Below £10,000 |  |
| --- | --- | --- | --- | --- |
| GCSE or O level |  |  | £10,001 to £30,000 |  |
| GCE, A-level or similar |  |  | £30,001 to £50,000 |  |
| Vocational (BTEC/NVQ/Diploma) |  |  | £50,001 to £70,000 |  |
| Bachelors degree or equivalent |  |  | Over £70,001 |  |
| Masters degree or equivalent |  |  | Prefer not to say |  |
| PhD, MD, or equivalent |  |  |  |  |
| Prefer not to say |  |  |  |  |

**8. Which of the following best describes you? 9. Religious faith**

| Asian or Asian British |  |  | None |  |
| --- | --- | --- | --- | --- |
| Black or Black British |  |  | Buddhist |  |
| Mixed |  |  | Christian/Catholic |  |
| White or White British |  |  | Hindu |  |
| Other ethnic group |  |  | Jewish |  |
| Prefer not to say |  |  | Muslim |  |
|  |  |  | Sikh |  |
|  |  |  | Other |  |
|  |  |  | Prefer not to say |  |
|  |  |  |  |  |

**9. CONTACT DETAILS**

***PLEASE PROVIDE YOUR CONTACT DETAILS SO WE CAN SEND YOU THE SECOND SURVEY IN 12 MONTHS’ TIME:***

***Name:***

***Email OR postal address (however you wish to receive the follow up survey):***

***Phone number :***

***We really appreciate you taking the time to take part in this study and would like to send you a £10 Amazon voucher as a token of our appreciation for your time. Please be aware that we cannot send you a voucher unless you have added your contact details above.***

***We understand that some people would prefer NOT to receive a voucher. If you would prefer not to receive the voucher, please tick this box:***

**If this survey has raised any questions for you about genomic testing, you can find further information from the following sources:**

Whole genome sequencing for a rare disease - Information for patients and family members

<https://www.england.nhs.uk/wp-content/uploads/2021/07/genome-sequencing-rare-disease-patient-information.pdf>

An easy read version of the same leaflet

<https://www.england.nhs.uk/wp-content/uploads/2021/07/genome-sequencing-rare-disease-patient-information-easy-read.pdf>

An animation about whole genome sequencing developed by researchers at Great Ormond Street Hospital

<https://tinyurl.com/genomictest>

Genomics England webpage with links and videos about genomic medicine

[https://www.genomicsengland.co.uk/genomic-medicine](https://eur01.safelinks.protection.outlook.com/?url=https%3A%2F%2Fwww.genomicsengland.co.uk%2Fgenomic-medicine&data=04%7C01%7Cb.friedrich%40ucl.ac.uk%7Ce592bbecaede4ccff37a08da13143b3e%7C1faf88fea9984c5b93c9210a11d9a5c2%7C0%7C0%7C637843274847804999%7CUnknown%7CTWFpbGZsb3d8eyJWIjoiMC4wLjAwMDAiLCJQIjoiV2luMzIiLCJBTiI6Ik1haWwiLCJXVCI6Mn0%3D%7C3000&sdata=eLxG%2Bl1CdCRNYKU7HRPLiYZgAkJ4EqFGdLhpDX1nrwA%3D&reserved=0)
